# The simplification of the insomnia severity index and epworth sleepiness scale using machine learning models

**DOI:** 10.1038/s41598-023-33474-8

**Published:** 2023-04-17

**Authors:** Woodo Lee, Hyejin Kim, Jaekwoun Shim, Dongsin Kim, Janghun Hyeon, Eunyeon Joo, Byung-Euk Joo, Junhyoung Oh

**Affiliations:** 1 Department of Physics, Korea University, Seoul, 02841 South Korea; 2 College of Pharmacy, Sookmyung Women’s University, Seoul, 04310 South Korea; 3 Institute of Educational Research, Korea University, Seoul, 02841 South Korea; 4 Sleep Research Center, NYX Corporation, Hanam, 12902 South Korea; 5 Semiconductor Research Institute, Korea University, Seoul, 02841 South Korea; 6 Department of Neurology, Samsung Medical Center, Seoul, 06351 South Korea; 7 Department of Neurology, Soonchunhyang University Seoul Hospital, Soonchunhyang University, Seoul, 31151 South Korea; 8 Institute for Business Research and Education, Korea University, Seoul, 02841 South Korea

**Keywords:** Sleep disorders, Machine learning

## Abstract

Insomnia and excessive daytime sleepiness (EDS) are the most common complaints in sleep clinics, and the cost of healthcare services associated with them have also increased significantly. Though the brief questionnaires such as the Insomnia Severity Index (ISI) and Epworth Sleepiness Scale (ESS) can be useful to assess insomnia and EDS, there are some limitations to apply for large numbers of patients. As the researches using the Internet of Things technology become more common, the need for the simplification of sleep questionnaires has been also growing. We aimed to simplify ISI and ESS using machine learning algorithms and deep neural networks with attention models. The medical records of 1,241 patients who examined polysomnography for insomnia or EDS were analyzed. All patients are classified into five groups according to the severity of insomnia and EDS. To develop the model, six machine learning algorithms were firstly applied. After going through normalization, the process with the CNN+ Attention model was applied. We classified a group with an accuracy of 93% even with only the results of 6 items (ISI1a, ISI1b, ISI3, ISI5, ESS4, ESS7). We simplified the sleep questionnaires with maintaining high accuracy by using machine learning models.

Insomnia, which causes serious disturbance in daily life, is one of the diseases showing a high prevalence in general populations. Not only does it augment such as headache and depression, but it can also impose a significant burden on healthcare costs^1–7^. Despite its high prevalence and significant morbidity, insomnia often remains unrecognized and untreated due to the difficulty of evaluation. For the assessment of insomnia, brief and valid questionnaires can be very useful because it takes less time and can be easily used by anyone. Currently, several patient-reported questionnaires are available for assessing insomnia symptoms, severities, correlates, and a variety of constructs presumed to contribute to the etiology of insomnia^8,9^. Among them, the Insomnia Severity Index (ISI) Table 1 is one of the most widely used.

**Table 1:** Insomnia severity index items (scores of each item is 0–4; 0 = no problem or difficulty; 4 = very severe problem).

Item no.	Question
1	Rate the current (i.e., last 2 weeks) severity of your insomnia problem(s)
1-a	Difficulty falling asleep
1-b	Difficulty staying asleep
1-c	Problems waking up too early
2	How satisfied/dissatisfied are you with your current sleep pattern?
3	To what extent do you consider your sleep problem to interfere with your daily functioning
4	How noticeable to others do you think your sleeping problem is in terms of impairing the quality of your life?
5	How worried/distressed are you about your current sleep problem?

It has been frequently used in numerous groups, including cancer patients, primary care patients, and veterans with Traumatic Brain Injury (TBI). Several studies demonstrated that the ISI had adequate internal consistency (Cronbach’s alpha = 0.74–0.92) by investigating the psychometric properties among diverse populations^10–15^. ISI items (Table 1) are rated on a 0-4 scale (5-point Likert scale), then all scores of 7 items are added up to make a total score of 0-28. It is known that the higher the ISI score, the more severe the symptoms of insomnia. The cut-off score for clinical insomnia on ISI is known as 15 points. On the other hand, excessive daytime sleepiness (EDS) is an inability to stay awake and alert during the major waking periods of the day, resulting in unintended lapses into drowsiness or sleep. EDS is a debilitating and potentially dangerous symptom that leads to poor productivity in daily life, including cognitive performance. It is also one of the most common complaints reported in sleep clinics and is often associated with various sleep disorders such as narcolepsy, obstructive sleep apnea, and periodic limb movement disorder. The ESS (Epworth Sleepiness Scale) (Table 2) is the representative questionnaire to assess subjective sleepiness and sleep propensity with important clinical utilization. ESS items (Table 2) are rated on a 0-3 scale (4-point Likert scale). Then, each item’s scores are added to make a total score ranging from 0 to 24.

**Table 2:** Epworth sleepiness scale items (score of each item is 0-3; 0 = would never doze; 3 = high chance of dozing).

Item no.	Situation
1	Sitting and reading
2	Watching TV
3	Sitting, inactive in a public place
4	As a passenger in a car for an hour without a break
5	Lying down to rest in the afternoon when circumstances permit
6	Sitting and talking to someone
7	Sitting quietly after a lunch without alcohol
8	In a car, while stopped for a few minutes in the traffic

While both the ISI and the ESS are the validated questionnaire tools for insomnia and EDS, it could be burdensome for patients to answer those measures repeatedly^16^. In addition, they are more compact and less accurate than the standard methods such as Polysomnography (PSG) and multiple sleep latency tests (MSLT) for assessing insomnia and EDS^17^.

Recently, as awareness and diagnosis of sleep disorders have increased, the cost of healthcare services associated with them have also increased significantly. These changes urge the healthcare system to be transformed from a hospital-centered system to a person-centered one, and ask for the development of tools to diagnose and track the main disease-related symptoms more efficiently and accurately.

With the developments of various sensors and devices, many healthcare researchers have been evaluated the Internet of Things (IoT) as high-potential research areas^18^. IoT technology in healthcare system can feature frequent measurements of representative health parameters for each disease and automatic records of abnormal events to collect information. If an accumulative set of measurements based on the IoT is used, to assess and track patient’s symptoms, which could not be obtained previously by a single visit to the clinic^19^, can become more feasible. Moreover, as IoT-based questionnaire development enables larger-scale data collection to be more faster and accurate, IoT-based questionnaire will promote big data research analysis. However, to simplify the questionnaire is essential above all for IoT-based questionnaire development. Because even questionnaires with less than ten items are occasionally cumbersome to use in healthcare facilities overflown with patients.

There have been several previous studies about the simplification of the ISI and modification of the ESS items. Table 3 demonstrates the related works. Researchers tried to simplify questions of the ISI, resulting in developments of the ISI-2 and ISI-3^16,20,21^. Even if some items of the ISI are excluded, the brief version must reflect both nocturnal and diurnal aspects of insomnia and detect insomnia with a significant discriminative validity. On the other hand, the same magnitude of simplification for the ESS has yet to be present, although some items were modified considering cultural differences among many countries^22–25^. Additionally, some researchers calculated alpha coefficients with a 7-item scale, after eliminating each of the questions one at a time. It means that removing one question did not affect strongly the internal consistency of the questionnaire^25^. The aforementioned studies demonstrated that the reliabilities of the ISI or ESS would not be affected seriously, even if some items of those questionnaires were deleted.

**Table 3:** Summary of previous researches about simplification of sleep questionnaire.

	Study and abbreviation of questionnaire	Items	Version
ISI	Wells et al.^20^: ISI-3	Items 2, 3, and 5	English
	Thakral et al.^21^: ISI-3	Items 2, 3, and 5	English
	Kraepelien et al.^16^: ISI-2	Items 2 and 3	English
ESS	Takegami et al.^25^: revised JESS	Replacing items 1 and 8 with new items	Japanese
	Rosales-Mayor et al.^23^: ESS-MPV	Repalcing item 8 with an alternative item	Spanish
	Zhang et al.^24^: mESS	Repalcing item 8 with an alternative item	Mandarin
etc	Nunzio et al.^26^: Berlin questionnaire	B1, (B6 or B7), D10	English

So, briefer tools with sufficient accuracy and reliability are needed for both patients and healthcare providers^8,14,27,28^. With this simplified instrument and IoT, those who need clinical interventions for sleep disorders could be distinguished earlier, leading a higher level of medical care and researches with a lower cost. For developing an optimized model, we have used machine learning, which makes data informative by extracting salient structures and classifying the data. ML makes it possible to make more powerful and accurate predictions, as various studies using ML have demonstrated^29,30^. Also, none of the existing work tried the simplified and combined form of the ISI and ESS for classifying people with sleep disorders within the best of our knowledge. Since the target disorders of the two questionnaires are different, our proposed model simultaneously classifies people with insomnia and/or daytime sleepiness. We expect that our model could help solve the inconvenience for people with sleep-wake disorders, as well as increase the accuracy of diagnostic decisions in clinical settings.

The main purpose of our study is to optimize the number of the ISI and ESS questionnaire items using machine learning algorithms and deep neural networks with an attention model. In this paper, we propose the novel simplified sleep questionnaire and machine learning algorithms with deep neural networks with remarkable efficiency and accuracy.

## Materials and method

### Subjects

We analyzed the medical records of 1,241 patients who got examined with PSG at Samsung medical center from January 2014 to May 2022. Eligible records that meet inclusion and exclusion criteria were 5,481 sets. A demographic analysis of the dataset is presented in Table 4.

- Inclusion criteria: Among those who got examined with PSG, people with insomnia or excessive daytime sleepiness as the chief complaints were included.
- Exclusion criteria: Among those who got examined with PSG, people who visited the clinic due to obstructive sleep apnea, restless leg syndrome, snoring, and rapid eye movement sleep behavior disorder were excluded from this research.

All experimental protocols were approved by The Institutional Review Board of Samsung Seoul Hospital, which also waived informed consent for this study (IRB no. 2022-07-003). The entire process of the study was performed in accordance with the ethical standards of the Declaration of Helsinki. Medical records including 6 surveys consisting of PSQI(Pittsburgh Sleep Quality Index), ISI, BDI (Beck depression inventory), ESS, SSS(Stanford Sleepiness Scale), and sleep habit questionnaire, as well as PSG data, were collected and evaluated.

**Table 4:** Demographic characteristics and clinical data.

	Median (IQR)	N
Demographic characteristics		
Age (years)	53 (40-62)	5481
Gender (M/F)	3892/1589 (71% M)	5481
Height (cm)	168 (161-174)	5481
Weight (kg)	71 (62-80)	5481
BMI (Body mass index)	25.1 (22.9-27.7)	5481
Clinical data		
Time in bed (min)	436 (403-467)	5481
Total sleep time (min)	362 (318-403)	5481
Sleep latency (min)	6.5 (3.0-13.0)	5481
Sleep efficiency (%)	85.6 (76.9-91.3)	5481
PSQI	7 (5-10)	4238
ISI	11 (6-15)	4338
ESS	9 (6-13)	4377
SSS	3 (2-3)	4365

### Exploratory data analysis

**Figure 1:**
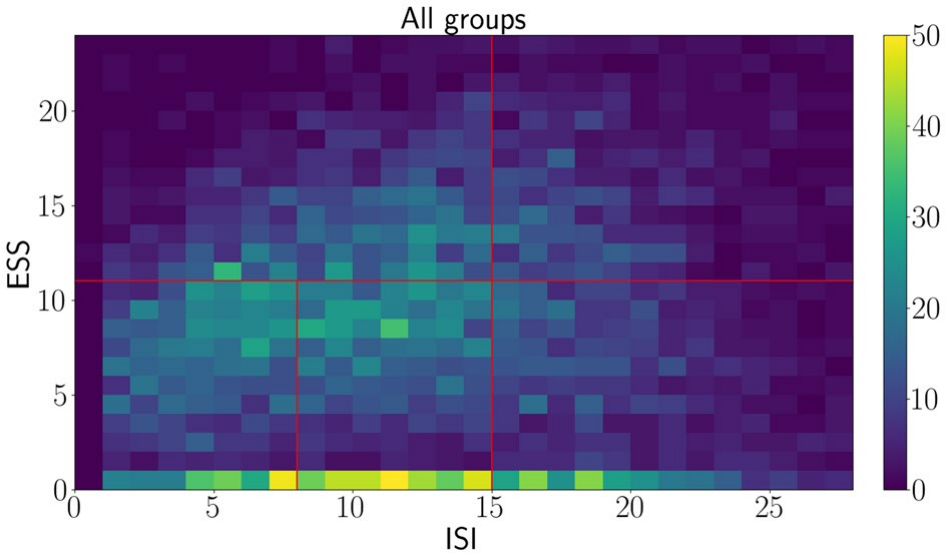
The distribution of total subjects.

All subjects are classified into five groups of their severity of insomnia and daytime sleepiness, and the number of subjects in the groups is different. The subjects in the dataset are classified into five groups based on Table 5, and the distribution of all subjects is shown in Fig. 1. The dataset for consisting questionary is built with the 5464 subject data, and the subjects are classified by the following Table 5 that shows the details of the subjects.

**Table 5:** The group notation table.

Groups	ISI cut-off scores (ISI)	ESS cut-off scores (ESS)
A	ISI $\le$ 14	ESS $\ge$ 11
B	ISI $\ge$ 15	ESS $\ge$ 11
C1	ISI $\le$ 7	ESS $\le$ 11
C2	8 $\le$ ISI $\le$ 14	ESS $\le$ 11
D	ISI $\ge$ 15	ESS $\le$ 11

Figure 1 shows a distribution of our dataset, and the red lines mean borderlines among five groups. Almost all the subjects answer zero for ESS questions, and the number of groups C1, C2, and D subjects is greater than that of groups A and B. More details for among groups are shown in the following Figures. The distribution also shows there are no distinctive patterns to distinguish among groups because the data dispersion is not clustered at any points. Mainly, the number of data is not even for among groups, and the imbalanced data could be an obstacle for classification with various machine learning algorithms.

**Figure 2:**
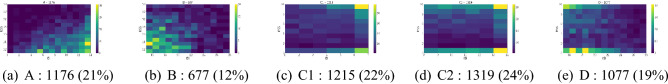
Histograms for among groups.

**Figure 3:**
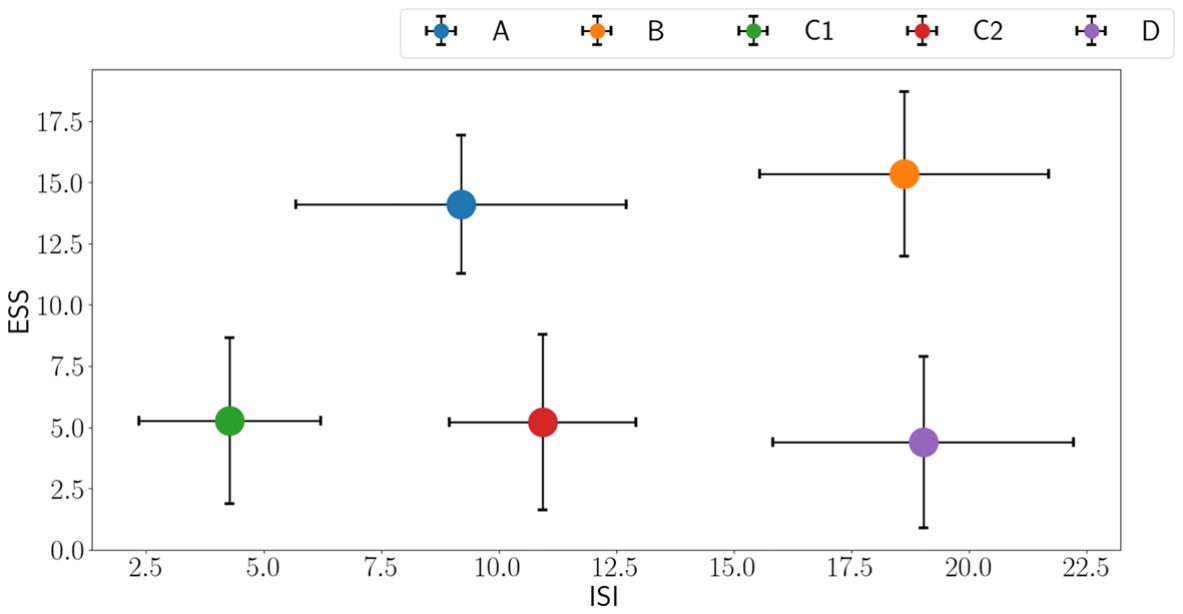
Distribution error bar plots with one $\sigma$ deviation.

Figure 2 shows the percentage of data and the number of subjects among groups. The subjects of group B are the smallest number, meaning there is little data on severe patients. C1 and C2 groups subjects are, for most of the dataset, showing that relatively less severe patients are the majority groups. The mean value with standard deviation is measured among groups to check our dataset’s uncertainty ranges. The uncertainty is defined by 1 $\sigma$, and the plots are shown in Fig. 3.

In a perspective ESS, the mean values of groups A and B are easily separated from C1, C2, and D. Note that groups A and B are heavy daytime sleepiness patients. However, given ISI, there are many overlapped uncertainty ranges among groups, which means ISI is not a prominent factor in classifying the groups. Classifying the groups with a strict rule is challenging due to the imbalanced data and randomly spread-out data. Accordingly, concise research questions in ESS and ISI, the ultimate goal for this research, is impossible to find golden rules with the dataset. Therefore, a machine learning-based classifier is developed to dig out the latent meanings that researchers could not find.

In addition, ESS and ISI data are analyzed independently to investigate different patterns. As mentioned in Section 1, ISI and ESS items have different characteristics, which could show different distributions.

**Figure 4:**
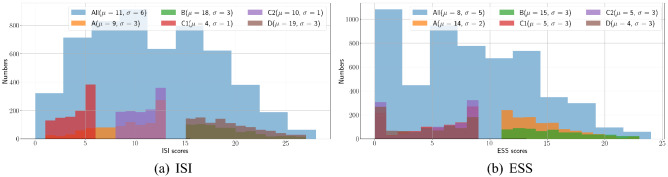
Histograms for each group, ISI and ESS.

Figure 4 shows that the distributions of ESS and ISI groups, and the mean value($\mu$) and standard deviation($\sigma$). The noticeable groups in Fig. 4a are B and D, who are suffered from insomnia. Their distributions of them are similar, and it means they are hard to be classified each other with ISI. In addition, the distribution of group A is broadly overlapped with groups C1 and C2, and group A is difficult to separate from groups C1 and C2. Figure 4b also shows more overlapped distribution than ISI distribution. Notably, groups C1, C2, and D are totally overlapped, and groups A and B are also overlapped. That is, ESS distribution could not be an essential factor for classifying subjects.

However, when considering both ISI and ESS distribution with independent items, the latent characteristics could be shown up. As a result, the ISI and ESS items could be simplified by sorting out essential and unimportant items.

### Development of the model

In this paper, traditional machine methods are first adopted to figure out what research questions and how many research questions are important to classify the subjects into five groups. We used 6 algorithms such as Decision Tree, Supporting Vector Machine, Extra Tree, Gradient Boost, K-nearest Neighbor, and Random Forest. These algorithms are selected to classify the dataset, and the input data is independent answers, not summation. There are 8 items in ESS section, 7 items in ISI section, and 15 input features in totally. 80% of data is used for training and the rest 20% of data is used for testing. Concerning for the imbalanced data, stratified splitting is considered. All algorithms are experiments for checking classification accuracy with excluding various combinations of research questions, and Fig. 6 shows the classification results. The total combination cases of excluded questions is 214,298, which are a huge numbers, and the results are checked out independently among cases. As mentioned above, the number of records is 5481, which is not enough to train machine learning algorithms. Due to the input features being a survey answer, digitized features and 1-D convolution layers are selected as an encoder. The encoder is expected to capture the latent patterns that could not be revealed in the summation (ISI, ESS) items. Moreover, the number of data (5464) is not enough size to use a huge model such as a transformer. In pursuit of a light version of deep neural nets, simple linear attention layers are selected to get the crucial items of the output tensor of the encoder. As a result, three layered 1-D convolution models with two layered attention models are built after experiments, and the model details are followed.

**Figure 5:**
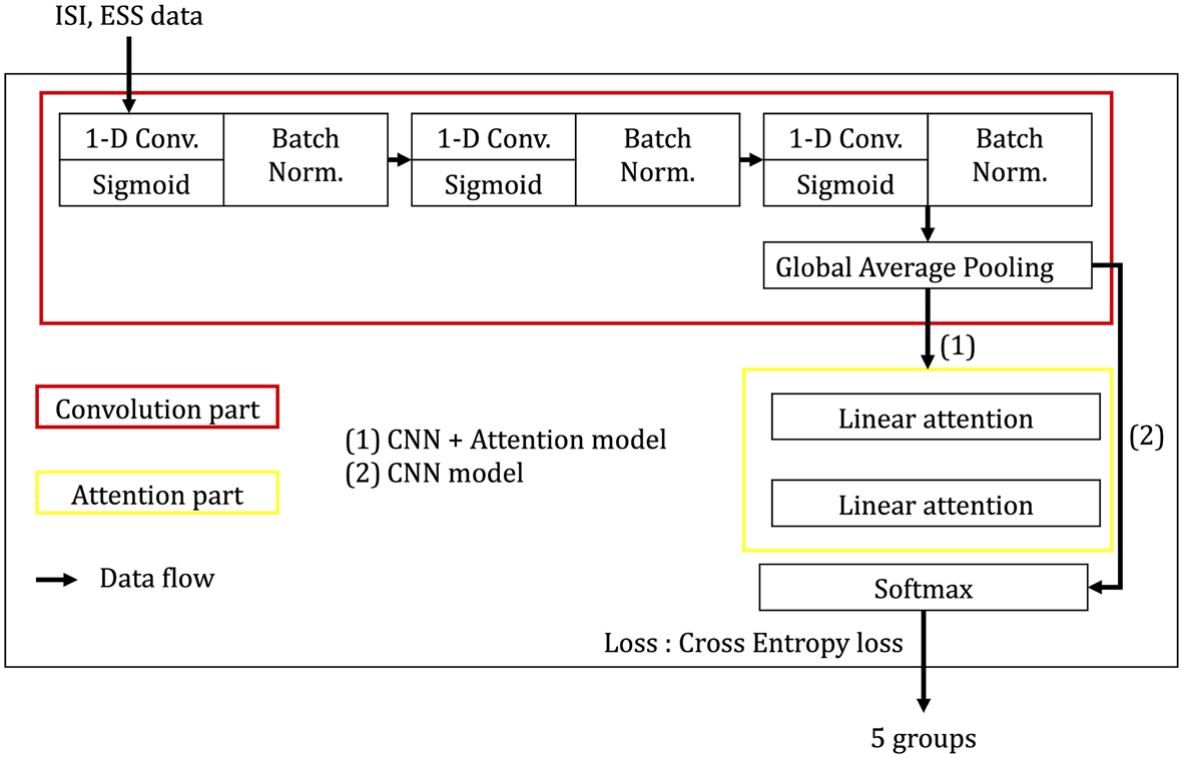
1-D CNN with attention model.

Figure 5 shows that our models and the first one-dimensional convolution layer catch the rough feature characteristics. The information of the output tensor from the first layer is entangled that cannot represent the detailed features of the data. In this sense, two more convolution layers are attached to catch the two more features independently. Note that the input data are formed by ESS and ISI groups. The second and third convolution layers have a role in separating ESS and ISI items that the model enables to classify the groups by learning the distinctive patterns for every five groups. Batch normalization (Batch Norm.) is adopted to prevent gradient vanishing. In addition, Batch Norm. can cover up the imbalanced features in embedding space. For example, the feature tensors would be clustered at some points accidentally when getting through the complex nets, and the tensor can be mismatched from their groups.

This normalization also prevents gradient exploding, and it helps over-fitting. Global average pooling layer is adopted to abstract 1-D vector for softmax layer for (1) CNN model, however, due to the drastic squeezing information, the model could not classify well with only CNN.

The most important part of our model is the last, which is the linear attention layers with the softmax layer in the case of (2) CNN+Attention model. The represented tensors are formed with two different groups, so two linear attention layers are applied before classifying by the softmax layer. The model could figure out the sensitive features by taking the protruded features from the attention layers. As the task is a classification, the cross-entropy loss function is selected.

We used accuracy, recall, precision, f1-score, and area under the curve (AUC) as the performance metrics for this model. The formulas for these metrics are as follows.

$$\begin{aligned} \text {Accuracy}= & {} \frac{\text {True Positives} + \text {True Negatives}}{\text {True Positives} + \text {False Positives} + \text {True Negatives} + \text {False Negatives}}\\ \text {Precision}= & {} \frac{\text {True Positives}}{\text {True Positives} + \text {False Positives}}\\ \text {Recall}= & {} \frac{\text {True Positives}}{\text {True Positives} + \text {False Negatives}} \\ \text {F1-Score}= & {} 2 \cdot \frac{\text {Precision} \cdot \text {Recall}}{\text {Precision} + \text {Recall}} \\ \text {AUC}= & {} \int _{-\infty }^{\infty } \text {TPR}(f)(\text {FPR}(f))' df \\ \text {TPR}(f)= & {} \frac{\text {TP}(f)}{\text {TP}(f) + \text {FN}(f)} \\ \text {FPR}(f)= & {} \frac{\text {FP}(f)}{\text {FP}(f) + \text {TN}(f)} \end{aligned}$$

## Results

Predicting and classifying the subjects’ groups with various modern algorithms show the availability of a computer science approach for medical data. Even though ambiguous medical data, such as questionnaire data, is hard to recognize, we could develop a model to distinguish the characteristics of each subject. Our compact deep-learning model successfully classifies the subjects into five groups with 15 features. Furthermore, we identified the most influential features based on our model and the medical information. As a result, we sorted out six questionnaire items that can aid medical research.

**Figure 6:**
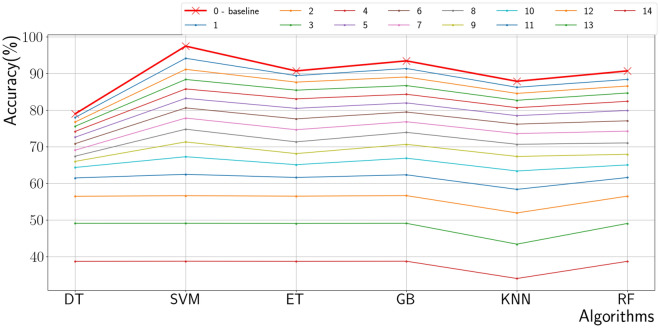
Machine learning-based classifier results.

A classical machine learning model provided a baseline accuracy of our study, which is shown in Table 6 and Fig. 6.

**Table 6:** Machine learning results with 6 ISI/ESS items.

Algorithm	Accuracy (%)
Decision tree (DT)	67
Supporting vector machine (SVM)	74
Extra tree (ET)	71
Grad boost (GB)	74
K-nearest neighbor (KNN)	70
Random forest (RF)	71

The plots on Fig. 6 are the average values for each excluded cases, and the Table 6 shows the results with 6 items. The results in Fig. 6 show that the accuracy is plunged when ten research questions are removed. Moreover, it is hard to lift up the accuracy with only the algorithms mentioned above. Even though they work well in our data, they cannot guarantee working well in the other data, and this is the limitation of traditional machine learning based on decision trees. Accordingly, the deep neural net-based algorithm is built, described in the net section. Before building deep neural net networks, the items should be selected to fix the input shape for networks. After discussing, selecting six items are reasonable to set a baseline for our research, and especially the following 3 cases could be significant, which is shown in Table 7.

**Table 7:** Case with 6 ISI/ESS items.

Case	Items
0	ESS7, ESS4
	ISI5, ISI1b, ISI3, ISI1a
1	ESS7, ESS2, ESS4
	ISI5, ISI1b, ISI3
2	ESS1, ESS4
	ISI5, ISI1b, ISI3 , ISI1a

To validate the attention layer’s effectiveness, we experiment with only using a 1-D convolution encoder as a part of the ablation study, which is shown in Table 8. The results in Table 9 shows that case 0 is the most important case among the 3 cases.

**Table 8:** 1-D CNN model results.

Case	Accuracy (%)	Precision (%)	Recall (%)	f1 score	Kappa coefficient	*p*
0	59	55	74	0.47	0.46	0.028
1	67	70	68	0.67	0.55	0.019
2	70	71	66	0.63	0.63	0.014

**Table 9:** 1-D CNN with attention model results.

Case	Accuracy (%)	Precision (%)	Recall (%)	f1 score	Kappa coefficient	*p*
0	93	94	94	0.93	0.78	<0.01
1	86	80	95	0.73	0.72	<0.01
2	75	72	87	0.63	0.65	0.01

Tables 8 and 9 show the attention part has a significant role in classifying the subjects into five classes with constricted features. The representation vector, the output of 1-D Conv. vectors, has 32 features that contain entanglement information from the six constricted features. Some of those are essential features, and some are not significant for classifying the groups. The attention part enhances the discernment of our model to focus on the critical features by raising the weight. Even though it is hard to recognize which features are empowered to make a decision in our model, it is clear that the performance of our model is improved by developing attention layers.

**Figure 7:**
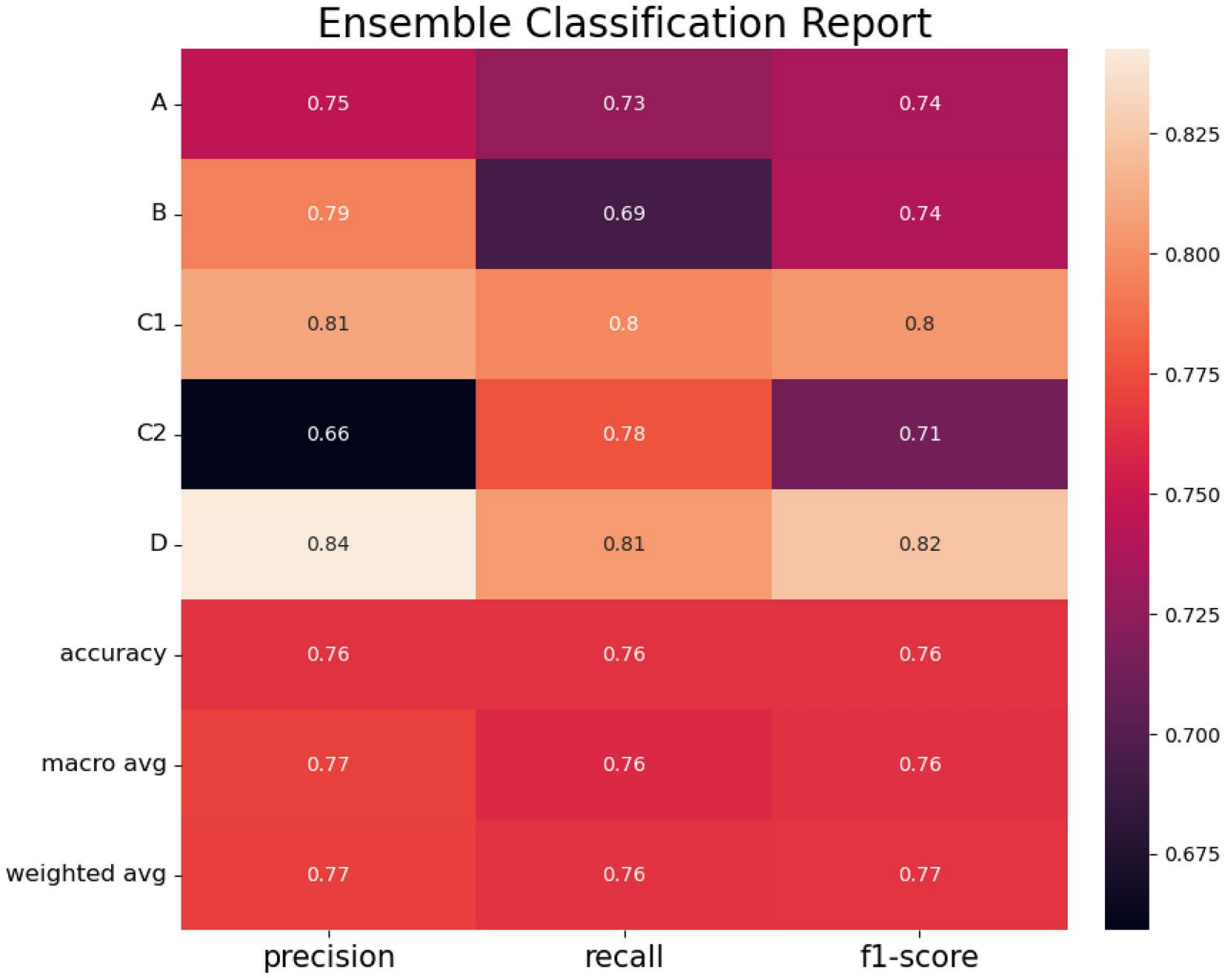
Ensemble classification report for 1-D CNN with attention model results.

**Table 10:** 1-D CNN with attention model’s AUC results.

	A	B	C1	C2	D
Case 0	0.73	0.94	0.92	0.63	0.93
Case 1	0.8	0.75	0.81	0.69	0.83
Case 2	0.69	0.68	0.67	0.63	0.73
Average case	0.74	0.79	0.8	0.65	0.83

Figure 7 shows the specific results for 1-D CNN with the attention model. Even though cases 0, 1, and 2 have different items, the average performance could be measured. Our model records a 0.77 f1-score, overwhelming the classical machine learning methods. Table 10 shows the AUC results for 1-D CNN with the attention model. Similar to our previous findings, case 1 performed the best.

## Discussion

We considered the following to create appropriate cases that reduced questionnaire items by more than 60%. Our three cases all include ISI1b, ISI3, and ISI5. Unlike previous research^16,20^, ISI2 was not included in our simplified model. Instead, ISI1a and/or ISI1b were included. Previous research on the simplification of ISI demonstrated that ISI2 and ISI3 differ from each other more prominently than the rest of the combinations. Therefore, we expected that the 2 items would be selected by the ML algorithms and DNN. This result can be explained by differences in the demographic characteristics of studies, for instance, ethnicity, present illness, or comorbidities.

There were examinations for differences in somnificities between items^31^. Except for pairs of ESS1-ESS4, ESS3-ESS7, and ESS6-ESS8, all the other pairs had significant differences. The result of ESS1-ESS4 was relatively complicated since the two items were not different between groups but significantly different among individuals. Unlike case 0 and case 1, case 2 includes ESS1 and ESS4, which showed the possibility of the same level of somnificities in the aforementioned research. This could affect the performance of distinguishing between people with EDS and ordinary people.

Although a brief version of the ESS has been absent, the redundancies of items 3, 7, and 6, 8, which were shown in the previous analysis of ESS questions^31,32^, could be helpful for effectiveness because the redundancies possibly exaggerate sleepiness due to double counting. In addition, item 8 caused frequent errors in translated versions. For example, the developer of the ESS mentioned that item 8 was about not only being at the wheel but also being a passenger in the car, but several Japanese versions of ESS mistakenly translated this question into ”while driving”. Therefore, ESS items in our 3 cases solved problems in conventional ESS^25^. Original ESS questions ask about daytime sleepiness in daily routine situations; however, some of the items could be unfamiliar to people with different lifestyles. The unfamiliarity mentioned above could account for the presence of missing data (up to 19.2% of participants in the ESS8)^25,33^. Because our model omits ESS8, it will reduce the rate of missing data.

In this way, three cases were created, and as a result of applying the artificial intelligence model to each case, case 0 showed high performance. Despite reducing the number of survey items (15 items to 6 items), it showed over 90% performance for all performance indicators. The simplified questionnaires are presented in Table 11. Therefore, selecting these survey items is appropriate and logical, and the experimental results also support the efficiency of the proposed method. However, despite this remarkable progress, there are some rooms to enhance accuracy further.

The first aspect is a classification model perspective, which enhances performance by adopting a better deep-learning model. The objective of this study is to reduce the number of questionnaire items while sustaining high accuracy. Therefore, the number of inputs for the model should vary with respect to reduced questionnaire items. To this end, we designed the proposed model based on the PointNet ^34^ architecture, which is a well-known classification method for unordered and unstructured datasets. However, since the PointNet-based method estimates each input independently and aggregates features using the symmetric function such as average-pooling or max-pooling, the local features (inter-class information between the inputs) are hard to extract, which limits the performances. To handle this problem, various methods ^35–37^ significantly improved the performances by proposing a method of aggregating neighboring information to extract better local features. Therefore, although the number of inputs is very different so it is difficult to apply them right away, it is expected that performance could be greatly improved by using the method of utilizing the inter-class information suggested by these methods.

The next aspect is the survey item optimization perspective, which might optimize survey items from a computer perspective. This study logically adopted three survey items from a human perspective. Nevertheless, there might have redundant information for the classification task among the selected items. Therefore, if you give different weights to each question and use dimension reduction using the machine learning method, it could find a better-optimized item set from a computer perspective.

**Table 11:** Questionnaire list simplified best as 6 items.

Item no.	Questionnaire
ISI 1-a	Rate the current severity of your insomnia problem of difficulty falling asleep
ISI 1-b	Rate the current severity of your insomnia problem of difficulty staying asleep
ISI 3	To what extent do you consider your sleep problem to interfere with your daily functioning?
ISI 5	How worried/distressed are you about your current sleep problem?
ESS 4	How likely are you to doze off or fall sleep as a passenger in a car for an hour without a break
ESS 7	How likely are you to doze off or fall sleep sitting quietly after a lunch without alcohol

## Conclusion and future directions

We propose a model that optimizes sleep questionnaire items using machine learning models and deep neural networks with attention models and verified its performance. Due to the change in times, the questionnaires need to be modified based on the objective standard of medical sciences and modern technology. In this sense, we showed the methods for brief questionnaires by designing machine learning models and deep neural networks with attention models. Finally, the 7 ISI and 8 ESS questions used to evaluate insomnia and EDS were simplified into a brief 6-questions questionnaire (ISI1a, ISI1b, ISI3, ISI5, ESS4, ESS7) to be available. By using machine learning models and deep neural networks with attention models, this new simple questionnaires showed 93% accuracy even with only 6 questions. This simplified questionnaire can enable to diagnose and track patients with insomnia and EDS more efficiently and accurately. Due to COVID-19 and the spread of IoT, the demand for remote medical monitoring has been increasing. Remote medical monitoring relies on the patient’s response, so it’s important to get the patient to respond accurately. By reducing the number of questions, we are able to increase the response rate and accuracy of the survey. Therefore, if this method is applied, data on other medical problems can be collected and analyzed more easily and accurately in addition to insomnia and EDS. Also, this will have great advantages in reducing health care costs.

## Data Availability

The data that support the findings of this study are available from NYX corporation but restrictions apply to the availability of these data, which were used under license for the current study, and so are not publicly available. Data are available from the authors upon reasonable request and with permission of NYX corporation(ceo@gosleep.kr).
